# Endoscopic Management of Foreign Bodies in the Gastrointestinal Tract: A Review of the Literature

**DOI:** 10.1155/2016/8520767

**Published:** 2016-10-11

**Authors:** Mikhael Bekkerman, Amit H. Sachdev, Javier Andrade, Yitzhak Twersky, Shahzad Iqbal

**Affiliations:** ^1^Lake Erie College of Osteopathic Medicine and St. John's Episcopal Hospital, Erie, PA, USA; ^2^SUNY Downstate Medical Center, Brooklyn, NY, USA; ^3^Department of Surgery, St. John's Episcopal Hospital, Far Rockaway, NY, USA; ^4^St. John's Episcopal Hospital, Far Rockaway, NY, USA

## Abstract

Foreign body ingestion is a common diagnosis that presents in emergency departments throughout the world. Distinct foreign bodies predispose to particular locations of impaction in the gastrointestinal tract, commonly meat boluses in the esophagus above a preexisting esophageal stricture or ring in adults and coins in children. Several other groups are at high risk of foreign body impaction, mentally handicapped individuals or those with psychiatric illness, abusers of drugs or alcohol, and the geriatric population. Patients with foreign body ingestion typically present with odynophagia, dysphagia, sensation of having an object stuck, chest pain, and nausea/vomiting. The majority of foreign bodies pass through the digestive system spontaneously without causing any harm, symptoms, or necessitating any further intervention. A well-documented clinical history and thorough physical exam is critical in making the diagnosis, if additional modalities are needed, a CT scan and diagnostic endoscopy are generally the preferred modalities. Various tools can be used to remove foreign bodies, and endoscopic treatment is safe and effective if performed by a skilled endoscopist.

## 1. Introduction

Foreign body ingestion is a common diagnosis that presents in emergency departments throughout the world. Food (typically meat) bolus impaction above a preexisting esophageal stricture or ring is by far the most common cause of esophageal foreign body obstruction in adults. Coins are the most common ingested foreign body in children. Three groups of people that are at a higher risk of ingesting foreign objects are children and adolescents, mentally handicapped patients or patients with psychiatric illness, and abusers of illicit drugs or alcohol [1]. Foreign body ingestion more commonly occurs in males, with some studies suggesting approximately a 1.5 : 1 male to female ratio [2, 3].

The most common types of foreign objects ingested (Table 1) differ between children and adults and each group may present with a unique set of symptoms [1, 3–5]. Upwards of 80% of foreign bodies pass spontaneously and do not require intervention [6], with less than 1% of all cases necessitating surgical intervention [7]. Despite the fact that most foreign bodies pass spontaneously, there is still significant morbidity and mortality associated with retained foreign bodies, with some reports estimating that nearly 1500 deaths occur in the United States annually due to foreign body ingestion [3]. The type of foreign body ingested may predispose patients to a particular site of impaction as well as common patterns of complications (see “Common Sites of Impaction of Sharp Objects Occur at Acute Angles or Intestinal Narrowing”). The upper esophagus is the most common lodgment site, followed by the middle esophagus, stomach, pharynx, lower esophagus, and finally the duodenum [3, 8].

*Common Sites of Impaction of Sharp Objects Occur at Acute Angles or Intestinal Narrowing*

 Sites of impaction
   Duodenal loop
   Duodenojejunal junction
   Appendix
   Terminal ileum

Symptoms regularly present as odynophagia, dysphagia, sensation of having an object stuck, chest pain, and nausea/vomiting in descending order [9, 10] (see “Clinical Presentations of Foreign Body Impaction”). Patients often can localize a site of discomfort; however, it is important to note that the site of discomfort does not correlate with the site of impaction on many cases [11].

*Clinical Presentations of Foreign Body Impaction*

 Symptoms
   Odynophagia
   Dysphagia
   Globus pharynges
   Chest pain
   Nausea/vomiting
   Abdominal pain

## 2. Epidemiology and the Types of Foreign Bodies

Children make up to 80% of patients that ingest foreign bodies, with 20% of all children between the ages of 1 and 3 having ingested some type of foreign body. Several studies have proposed that coins are the most frequently ingested foreign body in children [12, 13]. Button batteries are also commonly ingested foreign bodies in children, with one study estimating 2519 battery ingestion-related emergency department visits each year in children under 18 years of age [14]. As batteries come in multiple forms, they can predispose patients to distinct types of damage. Sodium or potassium hydroxide batteries can cause damage to the gastrointestinal mucosa through chemical burn, while lithium batteries likely damage tissues by eliciting an electric current through them [15]. Risk of complications from button battery ingestion is importantly associated with the size of the battery being >20 mm in diameter, children under 4 years of age, and length of time (>2 hours) in the gastrointestinal system [16]. Children have additionally been known to ingest toys, crayons, coins, and other objects found around the household.

Adults with psychiatric illnesses are also at an increased risk of foreign body ingestion, which can occur accidentally or intentionally, and many of these patients often present multiple times with recurrent foreign body ingestions [1]. Psychiatric patients frequently present after ingesting multiple ingested foreign bodies, as described by a case report where a 15-year-old male with mental retardation and psychiatric disorder was found to have 15 foreign bodies lodged in the stomach and lower esophagus [17] and in numerous other reports. Psychiatric patients have also been known to swallow foreign objects as a response to stress and a result of poor impulse control directed at their caregivers [18]. Incarcerated individuals may ingest foreign bodies as a method of obtaining secondary gain [19]. A careful history should be taken when assessing these patients due to increased risk associated with ingesting multiple foreign bodies.

Individuals under the influence of drugs and/or alcohol often present to emergency departments after ingesting multiple foreign bodies. The types of foreign bodies ingested tend to be spontaneous, and frequently patients do not remember swallowing the object [20]. There have been cases reported in the literature in which patients have ingested crack-cocaine pipes in an effort to evade detection by police [21, 22].

Accidental ingestion by adults, the geriatric population, and patients with decreased palate sensitivity is far less prevalent than in the aforementioned groups of individuals, but it does occur nevertheless with several notable patterns. 20% of adults that ingest foreign bodies do so while eating, and most foreign bodies discovered are from food boluses due to fish bone impaction [2, 3]. The most common esophageal foreign body in the western world is impacted food, and meat in particular [23]. In adults, a food bolus impaction is commonly due to an underlying structural abnormality such as eosinophilic esophagitis or a stricture. Less common etiologies include dentures, chicken bones, crab shells, wires, and bread bag clips [1, 24–26].

## 3. Complications of Foreign Body Impaction

The majority of foreign bodies pass through the digestive system spontaneously without causing any further harm, symptoms, or necessitating any further intervention [7]. Occasionally, complications will arise from ingested and impacted foreign bodies (Table 2). These complications are directly related to the type of foreign body and the location of impaction within the gastrointestinal tract.

A complication frequently reported associated with foreign body ingestion is intestinal perforation, which is predominantly caused by fish bones, yet <1% of foreign bodies are actually known to cause perforation. Perforations often present with erythema, crepitus, or tenderness. Fish bones are easily swallowed unnoticed and have sharp, pointed ends that predispose them to impaction at intestinal areas of acute angulation or narrowing, such as the duodenal loop, duodenojejunal junction, appendix, and ileocecal valve [1, 2]. Studies have shown that perforation most often occurs in the ileocecal region and colon, especially in the appendix and Meckel's diverticulum. Perforations in gastric and duodenal regions of the gastrointestinal tract are not encountered as frequently, and their presentations are more chronic and innocuous in nature [1, 2, 27]. Esophageal perforation has been reported at an incidence of 9.1% in patients with foreign body impaction of the esophagus [24]. Other foreign bodies known to cause perforation are animal bones from cow or chicken, crab shells, and wires [24]. Intestinal perforation further predisposes to hepatic abscess, sepsis, retroperitoneal hematoma, and hydronephrosis. One case report described a 61-year-old patient who presented with liver abscess after enterohepatic migration of an ingested fish bone [28].

Button batteries have been reported to cause chemical and electrical damage to mucosal tissues [15]. Beyond physical damage from the battery, complications have arisen where the battery was found to cause an esophageal stricture with the foreign body lodged in the esophagus surrounded by a mucus membrane, and, moreover, they have even been reported to pass through the esophageal wall and remain lodged within the mucosa [12]. Batteries can cause continuous injury for weeks in pediatric patients, predisposing them to aortoesophageal fistulas in addition to strictures [29].

Ingested objects that are larger in size and round in shape are able to lodge in the appendix and have an increased risk of causing appendicitis, appendiceal abscesses, and appendiceal perforation [1]. The prevalence of acute appendicitis due to foreign body ingestion is 0.0005% [13]. Appendicitis secondary to foreign object ingestion has been reported in cases of swallowed air gun pellets, razorblades, screws, and other metallic objects [30]. Rounded objects may lay dormant in the appendix for a long time asymptomatically and suddenly present as right lower quadrant abdominal pain years later, requiring surgical intervention [31].

## 4. Diagnosis

A thorough history is imperative in the diagnosis of foreign body ingestion and impaction. If history is unable to be obtained, as is the case of young children, psychiatric patients, or adults with physical limitations, a plain film radiograph of the chest and abdomen should be obtained. An initial radiographic assessment is usually the preferred initial step in foreign body management [32]. Radiographs can confirm the size, location, shape, and number of ingested foreign bodies [7]. However, many foreign bodies are radiolucent and plain films appear negative [1]. Objects that are opaque are typically made of glass, metals, animal bones (except for fish), and medications. It is important to note that aluminum, although it is a metal, is radiolucent on plain films. Objects such as most foods, fish bones, wood, and thorns are radiolucent.

If a patient is unable to provide a satisfactory history and radiography studies are negative, other modalities of diagnosis may be used. Computed tomography (CT) scanning and diagnostic endoscopy are generally the preferred modalities. CT scanning without contrast is superior to plain radiography and identifies foreign bodies in 80–100% of cases. Barium swallow studies are contraindicated in these patients due to possible mucosal perforation, and, likewise, these contrast agents may interfere with endoscopic evaluation. Therefore, a CT scan without contrast should generally be performed. The sensitivity of CT scan may be improved with 3D reconstruction [33]. After a CT scan is performed, endoscopic intervention can be performed.

## 5. Endoscopic Management and Surgical Intervention

If a patient is unable to pass a foreign body spontaneously, endoscopic intervention is recommended within 24 hours of ingestion [34]. The risk of complications associated with removal of foreign bodies is low and includes impaction, obstruction, and perforation [23, 35].

In managing patients with ingested foreign bodies, it is essential to assess the patient's airway. Patients that have increased secretions are at an increased risk and require urgent management. In some cases, endotracheal intubation is necessary and this is particularly beneficial in patients with proximal foreign bodies, patients who have ingested multiple objects, and patients with difficulty in removing foreign bodies [7]. The use of an overtube should also be considered to prevent an object from accidentally being dropped into the patient's airway. In addition, a laryngoscope should be immediately available in the event of airway obstruction.

The timing of upper endoscopy is variable and depends on the patient's age and the size, shape, and location of the foreign body. The American Society of Gastrointestinal Endoscopy has divided removal of foreign bodies into emergent endoscopic removal, urgent endoscopic removal, and nonurgent endoscopic removal [7] as follows.

*Timing of the Endoscopic Removal of Foreign Bodies*

 Timing of endoscopic removal is as follows.
   Emergent (immediate)
    (i) Esophageal obstruction
    (ii) Disk battery in the esophagus
    (iii) Sharp pointed objects in the esophagus
   Urgent (within 24 hours)
    (i) Esophageal objects that are not sharp and pointed
    (ii) Esophageal food impaction w/o complete obstruction
    (iii) Objects > 6 cm at or above the duodenum
    (iv) Magnets within endoscopic reach
   Nonurgent
    (i) Coins
    (ii) Objects in the stomach > 2.5 cm in diameter
    (iii) Disk batteries and cylindrical batteries in the stomach that can be observed up to 48 hours if asymptomatic (if longer than 48 hours, these batteries should be removed)

It is important to note that once a foreign body is in the stomach, the majority will pass within 4–6 days [7]. Conservative management has been proven to be effective in the management of many asymptomatic gastric foreign bodies [36, 37].

A number of endoscopic tools are available for foreign body removal and all endoscopists should be familiar with and comfortable using these tools. A flexible endoscope is important for both diagnosing and removing foreign bodies with a success rate of greater than 95% [8]. Flexible endoscopes are preferred when compared to rigid endoscopes because there is a lower risk of perforation [38]. Commonly used tools include polypectomy snares, grasping forceps, magnetic probes, retrieval snare net, and transparent cap-fitting device which is frequently used in endoscopic mucosal resection [39, 40]. An overtube is beneficial in that it protects the airway and facilitates passage of the endoscope to be more effective in piecemeal removal of a food impaction [41]. Depending on the type of impaction, different devices should be used.

For food boluses, and in patients with an underlying structural abnormality, patients often have complete obstruction and present with increased salivation. A snare or a snare basket is often the best device to use, and the foreign body can be removed in one piece or via a piecemeal extraction. After the bolus forms small pieces by a snare, it can be pushed into the stomach and can easy traverse the GI tract. It is important however to not push blindly as this can be extremely dangerous. Recently, however, two large studies have suggested that the push technique is in fact not associated with a higher risk of perforation in 375 patients. These studies suggest that gentle pressure applied to the middle of the food bolus and pushing the food bolus into the stomach may in fact be effective [42, 43]. It is important to note that the proteolytic enzyme such as papain, which enzymatically digests meat, should never be used because it can cause mucosal erosion and esophageal perforation. The administration of glucagon for esophageal food boluses (1 mg IV) has been recommended; however, it is controversial as to whether or not it is effective [44, 45]. Glucagon is relatively safe and may be used; however, endoscopic removal of the foreign body should not be delayed.

For short blunt objects, such as coins in children, approximately 30% of them will pass spontaneously within 24 hours [46]. Usually objects that are less than 2 cm in size can pass through the entire GI tract without causing any complications. If the objects however do not pass through the stomach after 3-4 weeks, they should be retrieved with a snare [8]. Foreign body forceps (rat tooth or alligator) or retrieval net can also be used [23, 47]. Objects wider than 2.5 cm may not easily pass the pylorus and therefore it is recommended that these objects be removed endoscopically; however, limited data is available to support this observation [8, 23].

For long objects, greater than 6 cm, such as toothbrushes and forks, spoons, or knives, endoscopic removal is recommended, as these are unlikely to pass through the duodenum. A snare or a basket should be used to remove these objects [19].

It is recommended that sharp pointed objects such as needles, nails, bread bag clips, toothpicks, and safety pins be removed before they pass into the stomach, as there is a high rate of perforation associated with sharp objects, usually near the ileocecal valve [7]. Otherwise, sharp pointed objects should be followed up with daily radiographs to document their passage [32]. Surgical intervention is often necessary if it appears that the patient has developed symptoms suggestive of a perforation, or if the sharp object has not progressed in a period longer than 72 hours [23]. Endoscopic removal can be achieved with a retrieval net, forceps, or a polypectomy snare [32]. In addition, for sharp objects, use of a condom-type hood or overtube is recommended. It is critical to remember that the sharp end should be a trailing point, as this will significantly reduce the risk of perforation [48].

For button batteries, it is important to remove these objects immediately after radiographic documentation if they are located in the esophagus due to the risk of perforation and esophageal burns. Batteries found in the stomach can be monitored for 48 hours unless the patient shows signs and symptoms of gastrointestinal injury, in which case urgent endoscopy should be performed [49]. During endoscopy, the use of a snare net or stone retrieval basket is advised [39]. Surgical management is recommended if severe abdominal pain is present.

For narcotic body packets (i.e., cocaine) that are smuggled across the border in protective coverings like condoms, it is important to not retrieve these endoscopically because if they puncture, death can result. These patients should be observed as inpatients, and surgery is indicated if there are signs of intestinal obstruction or clinical suspicion of rupture of the body packet [50].

For small bowel foreign bodies, single or double balloon enteroscopy can be used to gain access to the small intestine and remove such ingestions. Case reports have shown that this is an effective technique to remove retained video capsules and to remove objects that have high risk of perforation [51]. Hoods, forceps, and baskets can be used and have been specifically designed for enteroscopes.

## 6. Conclusions

Food bolus impaction above a preexisting esophageal stricture or ring is the most common cause of foreign body impaction in the western world. Most foreign bodies pass through the digestive system spontaneously without causing any further harm or necessitating any intervention. A thorough history, plain films, and 3D CT scans are useful in assessing patients with foreign body ingestion. Flexible endoscopy should be used for definitive treatment and timing of endoscopy varies depending on the type of foreign body ingested. Various tools can be used to remove foreign bodies and endoscopic treatment is safe and effective if performed by a skilled endoscopist.

## Figures and Tables

**Table 1:** Commonly ingested foreign objects.

Commonly ingested foreign bodies	Observed population
Coins	*Children*
Button batteries	
Crayons	
Toys	

Food boluses	*Adults*
Fish bones	
Chicken bones	
Dentures	
Crab shells	
Wires	
Pins	

**Table 2:** Common complications associated with foreign body impaction.

Foreign body	Complication
Button battery	Chemical/electrical damage, stricture formation, migration through intestinal wall
Fish bone	Perforation, peritonitis, abscess formation, sepsis, hematoma
Crack-cocaine pipes	Toxic effects of illicit drug
Bread bag clips	Attachment to bowel wall → inflammation, ulceration, perforation, obstruction
Round objects (air gun pellets, screws, other metallic objects)	Acute appendicitis
